# Supplementary material for: Morph-specific selection drives phenotypic divergence in color polymorphic tawny owls (Strix aluco) in Northern Europe
Source: Commun Biol. 2025 Dec 13;9:97. doi: 10.1038/s42003-025-09365-1 (PMC12827955; doi:10.1038/s42003-025-09365-1)
Supplement: Supplementary file 2 — Supplementary Information [file 42003_2025_9365_MOESM2_ESM.pdf]

Supplementary Information for:

Morph-specific selection drives phenotypic divergence in color polymorphic tawny owls (*Strix aluco*) in Northern Europe

Arianna Passarotto (0000-0002-6661-8714) [1,2,3,*,+],

Moritz D. Lürig (0000-0002-8175-6234) [1,4,+],

Esa Aaltonen [5],

Patrik Karell (0000-0003-0297-125X) [1]

[1] Evolutionary Ecology Unit, Department of Biology, Lund University, Lund, Sweden

[2] Universidad de Sevilla, Seville, Spain

[3] School of Biodiversity, One Health and Veterinary Medicine, University of Glasgow, Glasgow, Scotland, UK

[4] Florida Museum of Natural History, University of Florida, Gainesville, FL, USA

[5] Independent researcher, Lohja, Finland

[+] These authors contributed equally

*Corresponding author:
ariannapassarotto84@gmail.com

Tables

Supplementary Table 1 - Breakdown of the number of observations. The removal of incomplete observations (intersection of all individuals with complete ID records and a standard deviation (SD) for color score that was smaller than 1.5 [see Supplementary Fig. 1]) left us with 1972 usable records from 957 adult breeding individuals and 206 recruits. 68% of the observations (67% of the individuals) in the usable dataset were gray morphs. Due to missing covariates (e.g., laying date and/or both parent ID) we could not use all observations in the structural equation models (SEMs).

	**Observations**	**Individuals**
All adults in dataset	2318	1037 + X
With ring ID	2132	1037
Color score SD <= 1.5	2077	1018
Color score median = 9	1985	962
Year >= 1980	1972	957
All recruits in dataset	219	219
With morph / color score information	206	206
Gray morph adults	1312	623
Brown morph adults	660	334
Gray morph recruits	149	149
Brown morph recruits	58	58
SEM 1 (obs. w. complete information)	1866	882
SEM 2 (obs. w. complete information)	170	170

Supplementary Table 2 - Summary tables for supplementary generalized additive models. Non-linear tests (Generalized Additive Models, GAMs) for differences between morphs in color score dynamics of the entire population and immigrants only (*N* = 466; GAM S1a), the entire population and the new cohort of every year (*N* = 957; GAM S1b); as well as differences between morphs in temporal dynamics of laying date (*N* = 1878; GAM S2) and recruitment success (*N* = 1972; GAM S3) of breeding adults.

Model	Response variable	Component	Term		Est. df	Ref. df	Chi.sq / F-value	p-value
GAM S1a	Color score (only immigrants)	Linear component		morph		1	0.564	0.453
		Smooth terms	Year	gray	3.814	3.976	5.371	0.0003
				brown	1.241	1.43	3.054	0.1271
			Observer	gray	1.697	2	16.31	< 0.0001
				brown	1.826	2	5.371	0.0024
GAM S1b	Color score (only new cohort)	Linear component		morph		1	0.061	0.805
		Smooth terms	Year	gray	3.471	3.846	23.685	< 0.0001
				brown	1.001	1.002	7.207	0.0074
			Observer	gray	1.928	2	58.202	< 0.0001
				brown	0.421	2	0.293	0.2395
GAM S2	Laying date	Linear component		morph		1	0.774	0.379
		Smooth terms	Year	gray	3.943	3.998	28.64	< 0.0001
				brown	3.863	3.988	16.67	< 0.0001
GAM S3	Recruitment success	Linear component		morph		1	0.523	0.47
		Smooth terms	Year	gray	2.596	3.114	10.800	0.0163
				brown	2.337	2.845	3.000	0.2632

Supplementary Table 3 - Summary table for supplementary linear models. Linear models (LMs) 1-3 are counterparts to GAMs 1, S2, and S3, testing for temporal dynamics of color scores, laying date and recruitment success in a linear fashion (correspond to dashed lines in Fig. 2A, C and D).

Model	Response variable	Term	Df	Deviance	Resid. Df	Resid. Dev	F-value	p-value
LM 1	Color score	Year	1	64.385	1970	2321.8	56.001	< 0.0001
		Morph	1	1.142	1969	2320.7	0.993	0.3191
		Year × morph	1	58.059	1968	2262.7	50.499	< 0.0001
LM 2	Laying date	Year	1	9842.4	1876	301251	61.271	< 0.0001
		Morph	1	196.9	1875	301054	1.226	0.2684
		Year × morph	1	19.7	1874	301034	0.123	0.7260
LM 3	Recruitment success	Year	1	7.5314	1970	1701.4	-	0.0061
		Morph	1	0.3945	1969	1701	-	0.5299
		Year × morph	1	1.9139	1968	1699.1	-	0.1665

Supplementary Table 4 – Results for heritability analyses. Parent-offspring regression (POR) model estimating heritability from midparent-offspring resemblance and the two animal models (AM1–2) using pedigree-derived relatedness to partition phenotypic variance into additive and dominance components with associated estimates and standard error (SE). For AM1 and AM2 variance components are also provided, showing additive genetic, shared nest effects and residual variance estimates with associated Z-ratios, and AIC for model comparison.

Model			Estimate	SE	t value	p-value	Var. comp.	SE	Z-ratio	AIC
POR	Fixed effects	Intercept	1.597	0.618	2.584	0.0106	-	-	-	-
		Midparent color score	0.825	0.076	10.797	< 0.0001	-	-	-	-
AM 1	Fixed effects	Intercept	8.038	0.271	29.70	<0.0001	-	-	-	-
	Random effects	Additive genetic	-	-	-	-	7.379	1.683	4.383	176.714
		Common environment (Nest ID)	-	-	-	-	0.108	0.601	0.180	
		Residual variance	-	-	-	-	0.763	1.246	0.612	
AM 2	Fixed effects	Intercept	4.884	1.680	2.907	0.0036	-	-	-	-
		Additive genetic effect	2.279	0.325	7.015	<0.0001	-	-	-	-
		Dominance genetic effect	0.588	0.433	1.356	0.1750	-	-	-	-
		Mean breeding snow depth	0.002	0.041	0.038	0.9693	-	-	-	-
		Number of breeding snow days	-0.037	0.047	-0.781	0.4347	-	-	-	-
		Mean breeding air temperature	-0.093	0.126	-0.733	0.4635	-	-	-	-
		CV of breeding air temperature	4.528	2.412	1.877	0.0605	-	-	-	-
	Random effects	Additive genetic	-	-	-	-	1.260	1.067	1.181	100.659
		Common environment (Nest ID)	-	-	-	-	0.523	0.711	0.736	
		Residual variance	-	-	-	-	3.674	1.205	3.049	

Supplementary Table 5 – Results for heritability analyses. Generalized least squares (GLS) models based on POR and AM2 outputs, testing for changes in the residuals of POR and AM2 over time, respectively.

**Model**	**Fixed effect**	**Estimate**	**Std. error**	**t value**	**p-value**
GLS POR	Intercept	-0.032	0.376	-0.085	0.9321
	Year	-0.057	0.018	-3.137	0.0020
	Morph	3.012	0.663	4.542	< 0.0001
	Year × morph	0.035	0.030	1.157	0.2490
GLS AM2	Intercept	0.082	0.234	0.353	0.7245
	Year	-0.038	0.011	-3.595	0.0004
	Morph	1.676	0.510	3.289	0.0012
	Year × morph	0.033	0.021	1.548	0.1235

Supplementary Table 6 - Summary table for temporal trends in linear models. Trends for linear temporal dynamics (i.e., coefficients - correspond to dashed lines in Fig.2A, C and D).

Model	Morph	Trend	SE	Df	Lower CL	Upper CL	t-ratio	p-value
LM 1	gray	-0.028	0.003	1968	-0.033	-0.022	-10.076	< 0.0001
	brown	0.006	0.004	1968	-0.001	0.014	1.608	0.1080
LM 2	gray	-0.202	0.033	1874	-0.267	-0.136	-6.043	< 0.0001
	brown	-0.222	0.047	1874	-0.315	-0.129	-4.687	< 0.0001
LM 3	gray	-0.021	0.007	-	-0.035	-0.007	-2.929	0.0034
	brown	-0.004	0.010	-	-0.024	0.016	-0.360	0.7191

Supplementary Table 7 - Summary table for the Structural Equation Model (SEM) in breeding adults. SEM 1 tested for environmental effects on breeding adult color score, laying date and recruitment success across both morphs (*N* = 1866). 95% confidence intervals (CI) are also provided.

Response	Term	Estimate	Estimate (std.)	Std. Error (std.)	z-value (std.)	p-value (std.)	CI lower	CI upper
Air temp. (Mean)	Year	0.379	0.379	0.018	21.055	< 0.0001	0.343	0.414
Air temp. (CV)	Year	-0.071	-0.071	0.022	-3.153	0.0016	-0.114	-0.027
Snow depth (Mean)	Year	0.063	0.063	0.028	2.273	0.0230	0.009	0.117
Snow days (N)	Year	-0.426	-0.426	0.021	-20.736	< 0.0001	-0.466	-0.385
Color score	Air temp. (Mean)	0.001	0.001	0.041	0.025	0.9798	-0.079	0.081
	Air temp. (CV)	-0.008	-0.008	0.026	-0.313	0.7542	-0.059	0.043
	Snow depth (Mean)	-0.089	-0.088	0.029	-3.079	0.0021	-0.145	-0.032
	Snow days (N)	0.081	0.081	0.043	1.880	0.0601	-0.003	0.165
	Morph	-0.087	-0.041	0.023	-1.796	0.0724	-0.085	0.004
Laying date	Air temp. (Mean)	-0.246	-0.253	0.041	-6.207	< 0.0001	-0.333	-0.173
	Air temp. (CV)	-0.005	-0.005	0.019	-0.256	0.7982	-0.043	0.033
	Snow depth (Mean)	0.083	0.085	0.024	3.534	0.0004	0.038	0.133
	Snow days (N)	0.088	0.090	0.042	2.172	0.0298	0.009	0.172
	Color score	0.028	0.029	0.022	1.299	0.1941	-0.015	0.072
	Morph	-0.076	-0.037	0.022	-1.689	0.0913	-0.080	0.006
Recruitment success	Air temp. (Mean)	-0.002	-0.005	0.043	-0.115	0.9087	-0.088	0.079
	Air temp. (CV)	0.012	0.033	0.027	1.235	0.2170	-0.019	0.086
	Snow depth (Mean)	-0.022	-0.060	0.023	-2.610	0.0090	-0.105	-0.015
	Snow days (N)	0.009	0.023	0.043	0.541	0.5888	-0.061	0.108
	Color score	-0.0004	-0.001	0.021	-0.052	0.9584	-0.043	0.041
	Laying date	-0.009	-0.025	0.023	-1.056	0.2911	-0.071	0.021
	Morph	-0.015	-0.019	0.023	-0.854	0.3933	-0.064	0.025

Supplementary Table 8 - Summary table for the Structural Equation Models (SEM) in recruits. SEM 2 tested for genetic and environmental effects in the recruits (*N* = 170) through path analysis (see Fig. 4A and 5A). 95% confidence intervals (CI) are also provided.

Response	Term	Estimate	Estimate (std.)	Std. Error (std.)	z-value (std.)	p-value (std.)	CI lower	CI upper
Paternal color score	Paternal morph	-0.062	-0.031	0.077	-0.406	0.6846	-0.181	0.119
Maternal color score	Maternal morph	0.172	0.084	0.076	1.101	0.2707	-0.065	0.233
Laying date	Paternal color score	0.209	0.212	0.071	2.991	0.0028	0.073	0.351
	Maternal color score	-0.127	-0.123	0.072	-1.709	0.0875	-0.264	0.018
	Paternal morph	-0.157	-0.080	0.072	-1.105	0.2691	-0.221	0.062
	Maternal morph	-0.404	-0.192	0.071	-2.710	0.0067	-0.331	-0.053
Air temp. breeding (Mean)	Laying date	0.864	0.870	0.019	46.866	< 0.0001	0.832	0.906
Air temp. breeding (CV)	Laying date	-0.256	-0.272	0.071	-3.838	0.0001	-0.413	-0.135
Snow depth breeding (Mean)	Laying date	-0.415	-0.416	0.063	-6.570	< 0.0001	-0.542	-0.294
Snow days breeding (N)	Laying date	-0.718	-0.655	0.044	-14.973	< 0.0001	-0.741	-0.570
Recruit morph	Paternal morph	0.532	0.558	0.046	12.246	< 0.0001	0.469	0.647
	Maternal morph	0.291	0.286	0.056	5.130	< 0.0001	0.176	0.395
Recruit color score	Recruit morph	-0.254	-0.117	0.071	-1.653	0.0971	-0.256	0.021
	Paternal color score	0.161	0.155	0.072	2.164	0.0300	0.015	0.295
	Maternal color score	0.329	0.305	0.068	4.466	< 0.0001	0.171	0.439
	Air temp. breeding (Mean)	0.043	0.041	0.092	0.449	0.6759	-0.141	0.218
	Air temp. breeding (CV)	0.136	0.122	0.073	1.673	0.0935	-0.021	0.264
	Snow depth breeding (Mean)	-0.041	-0.039	0.076	-0.512	0.6121	-0.188	0.111
	Snow days breeding (N)	-0.005	-0.005	0.087	-0.056	0.9488	-0.176	0.165

Figures

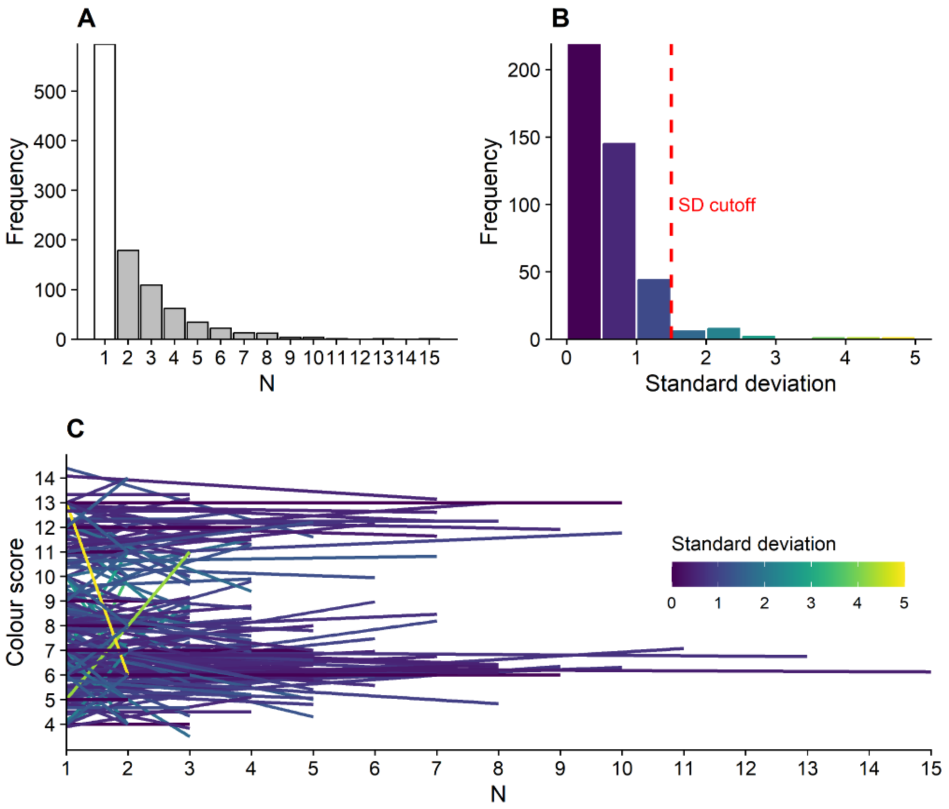

Supplementary Figure 1 - Repeatability of color scoring per individual. A: Number of measurements per individual over the years. **B:** Distribution of variation of color scores of individuals that were measured at least two times (gray bars in panel A). We used standard deviation (SD) to quantify the spread of multiple measurements around an individual's mean, reflecting the uncertainty in estimating that mean. Individuals with a standard deviation higher than 1.5 were not used in the analyses (dashed red bar indicates cut-off). **C:** Regression lines of color scores of individuals that were measured multiple times (including the ones that were not used). The color coding denotes the SD across all measurements per individual.

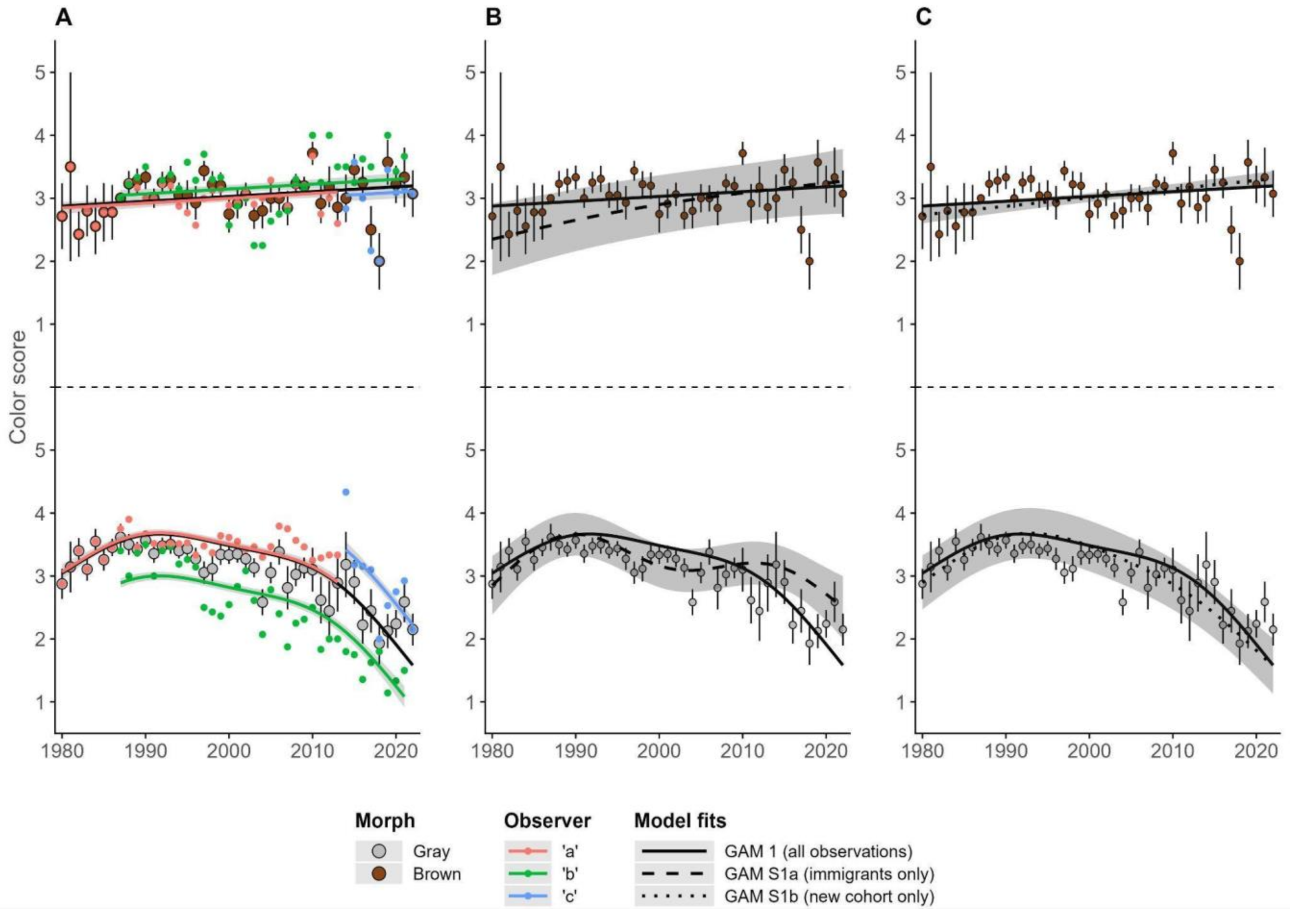

Supplementary Figure 2 - Relative color score dynamics - observer effects and demographic contexts. A: Large points denote the yearly average of morph-specific color scores using all individuals per each year (mean ± SE, $N = 1972$); small three-colored points indicate observer specific averages per year. The solid black line is the model fit from GAM 1 (shading denotes ± CI of fit) with transformed estimates (gray + 3, brown + 9), colored solid lines are fits for observer random intercept effects. **B:** Yearly average (mean ± SE) of morph-specific color scores contrasting the entire population (black solid line) and immigrants (black dashed line; $N = 466$). Model fit for immigrants stems from GAM S1a; shading denotes ± CI of fit. **C:** Yearly average (mean ± SE) of morph-specific color scores contrasting the entire population (black solid line) and new breeding cohorts for each year (black dotted line; $N = 957$). Model fit for new cohorts stems from GAM S1b; shading denotes ± CI of fit.

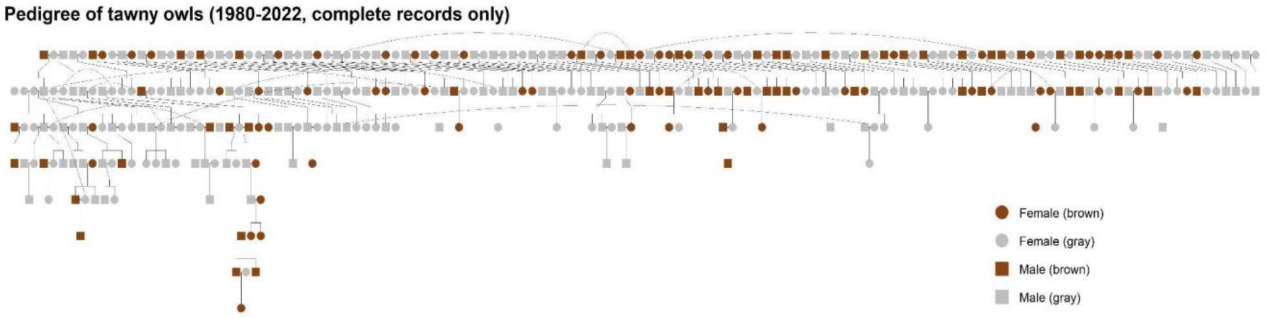

Supplementary Figure 3 - Pedigree of tawny owls. Visualized pedigree used in the parent-offspring regression (Fig. 3; Supplementary Table 3) and the animal models (complete records: $N = 170$). The pedigree is highly skewed, with only few recruits per dam (1.62 ± 0.09 recruits per dam, mean ± SE).

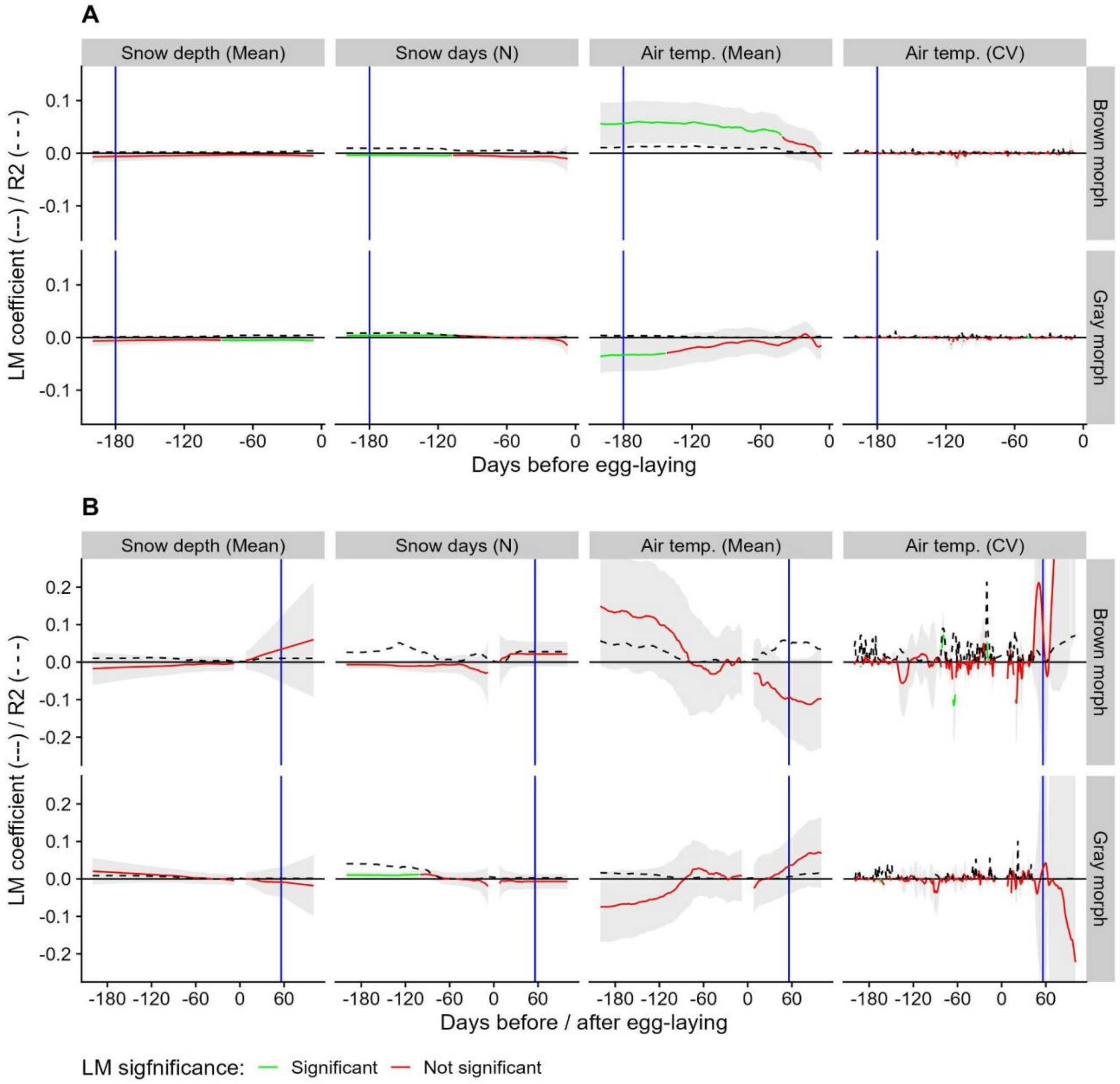

Supplementary Figure 4 - Sensitivity-analysis of linear model outcomes to the choice of time window for climate predictors. Model coefficients (solid lines) and R² values (dashed lines) from linear regressions of differently sized windows, testing **A:** the association between winter conditions (x-axis: number of days before egg-laying, i.e., prior the onset of breeding period) and adult plumage color scores, analyzed separately by morph (brown vs gray), and **B:** Same analysis for juvenile plumage color scores (recruits), with days counted both before and after hatching (egg-laying + 30 days). Columns represent different climate metrics: mean snow depth, number of snow days, mean air temperature, and temperature variability (CV). Lines are colored by model significance (green = $p < 0.05$, red = $p \geq 0.05$); shaded areas denote 95% CI of coefficient estimates. Vertical blue lines indicate the windows used in the final path analysis for adults (SEM1, 180 days), and for recruits (SEM2, 56 days).
